# Comparison of Experimental Pain Modulation by Hypnosis, Virtual Reality and Virtual Reality Hypnosis in Healthy Individuals

**DOI:** 10.1002/ejp.70166

**Published:** 2025-11-10

**Authors:** Floriane Rousseaux, Émilie Giguère, Mathieu Landry, Mathieu Piché, Maedeh Mostanadi, Pierre Augier, Jessica Simon, Aminata Bicego, Marie‐Elisabeth Faymonville, Anne‐Sophie Nyssen, David Ogez, Pierre Rainville, Audrey Vanhaudenhuyse

**Affiliations:** ^1^ Conscious Care Lab, GIGA Consciousness, GIGA Institute University of Liège Liège Belgium; ^2^ Laboratory of Cognitive Ergonomics and Work Intervention University of Liège Liège Belgium; ^3^ Medical Hypnosis Laboratory Research Center of Maisonneuve‐Rosemont Hospital Montreal Québec Canada; ^4^ Research Centre, Institut Universitaire de Gériatrie, CRIUGM University of Montreal Montreal Québec Canada; ^5^ Laboratory of Social Neuroscience and Electrophysiology (LENS) University of Montreal Montreal Québec Canada; ^6^ Cognitive and Computational Neuroscience Laboratory (CoCoLab) University of Montreal Montreal Québec Canada; ^7^ McGill University Montreal Québec Canada; ^8^ Département d'Anatomie Université du Québec à Trois‐Rivières Trois‐Rivières Québec Canada; ^9^ Psychology and Neuroscience of Cognition University of Liège Liège Belgium; ^10^ Oncology Integrated Arsene Bury Center University Hospital of Liège Liège Belgium; ^11^ Département de Stomatologie University of Montreal Montreal Québec Canada; ^12^ Algology Interdisciplinary Centre University Hospital of Liège Liège Belgium

**Keywords:** acute pain, experimental pain, hypnosis, nociceptive flexion reflex, virtual reality, virtual reality hypnosis

## Abstract

**Background:**

Hypnosis (H) and virtual reality (VR) are effective behavioural interventions to influence acute pain perception. Hypnotic suggestions have also been shown to modulate the nociceptive flexion reflex (NFR), suggesting the activation of descending modulatory mechanisms affecting spinal nociceptive activity. The combination of these techniques, virtual reality hypnosis (VRH), may reduce pain, but research on their comparative efficacy and mechanisms requires further experimental investigation. This study compared the effects of relaxation hypnosis, VR and VRH on pain perception and nociceptive physiological responses.

**Methods:**

Twenty‐four healthy participants were tested at baseline followed by three experimental conditions (relaxation hypnosis, VR, VRH) in a counterbalanced order. Pain intensity and unpleasantness, as well as NFR amplitude evoked by noxious transcutaneous electrical stimulation, were measured. Bayesian statistics assessed evidence for analgesic effects on each variable.

**Results:**

The strength of evidence in favour of our hypotheses was categorised as follow: BF = 1–3: anecdotal evidence; BF = 3–10: moderate evidence and BF > 10: strong evidence. For NFR values, Bayesian paired‐sample T‐tests provided anecdotal support for the efficacy of relaxation hypnosis (BF + 0 = 2.11) and stronger evidence for VR (BF + 0 = 10.94) and VRH (BF + 0 = 14). For pain intensity, moderate evidence supported reductions with relaxation hypnosis (BF + 0 = 9.18), while strong evidence was found for VR (BF + 0 = 27.99) and low to moderate for VRH (BF + 0 = 5.88). Similarly, unpleasantness showed anecdotal reduction with hypnosis (BF + 0 = 1.9), and moderate evidence supported VR (BF + 0 = 4.86) and VRH (BF + 0 = 7.18). Across all measures, no significant differences were found between hypnosis, VR and VRH.

**Conclusion:**

These findings suggest that these techniques did not differentially affect NFR, pain intensity, or unpleasantness.

**Significance Statement:**

The strength of this fundamental study is to directly compare hypnosis, VR, and VRH on both pain perception and physiological responses. It shows that VR alone is effective, while adding hypnosis does not always lead to better results and the combination could even create interference in some cases. This article helps to nuance the existing literature and common assumptions about the tool's use. These findings help clarify how VRH works and to propose guidance for clinical practices and further VRH development.

## 1 Introduction

Pain is a complex biopsychosocial phenomenon characterised by sensory, affective and cognitive dimensions, serving as an essential nociceptive alarm system for potential or actual tissue damage (IASP 2020; Raja et al. 2020). Given pain's multidimensional nature, integrative assessment methods are necessary. The current study focuses on the management of experimentally induced acute pain. In this context, acute pain serves as a controlled model for investigating fundamental pain mechanisms. The biopsychosocial model conceptualises pain as an interplay between biological factors, psychological components and social influences. Effective management necessitates addressing both neurophysiological and psychological mechanisms to alleviate suffering. Although the biopsychosocial framework is more commonly applied to chronic conditions, understanding how psychological and physiological factors interact in acute pain models remains essential for developing effective interventions (Jensen et al. 2015; Rainville et al. 1997). Objective measures, such as nociceptive responses, provide physiological insights, whereas validated subjective scales help capture the participant's perceptual and emotional experience. To investigate pain processing at spinal and supraspinal levels, assessing nociceptive activity and descending modulation alongside subjective evaluations is essential (Sandrini et al. 2005). The nociceptive flexion reflex (NFR), reflecting spinal nociceptive activity independent of supraspinal input, has been widely used for this purpose (Willer and Bathien 1977; Sandrini et al. 2005). Reflex amplitude generally correlates with pain intensity (Willer and Bathien 1977; Chan and Dallaire 1989; Guieu et al. 1992). Integrative interventions such as hypnosis or VR have shown efficacy in pain modulation (Ferguson et al. 2020; Rousseaux et al. 2020; Bicego et al. 2021). Hypnotic suggestions can alter the amplitude of the reflex, suggesting that hypnotic states can directly affect pain processing at the spinal level (Houzé et al. 2021; Danziger et al. 1998; Kiernan et al. 1995). *Hypnosis* can be defined as ‘a state of consciousness involving focused attention and reduced peripheral awareness, characterised by an enhanced capacity for response to suggestion’ (Elkins et al. 2015). Key components of hypnosis include absorption, dissociation, bodily awareness, automaticity (Spiegel 1991; Weitzenhoffer 2002). Hypnosis is used for managing acute and chronic pain, reducing pain intensity and unpleasantness (Bicego et al. 2022; Kumar Govindaiah et al. 2024; Elkins et al. 2015). *Virtual reality (VR)* is described as an immersive technology that creates a strong sense of presence in a computer‐generated environment (Slater and Sanchez‐Vives 2016, 4). VR alone has shown promise in pain modulation by providing immersive, multisensory distractions (Hoffman et al. 2000). By combining these modalities, *virtual reality hypnosis (VRH)* could leverage the immersive properties of VR alongside the cognitive modulation of hypnosis, potentially offering additive or synergistic effects (Patterson et al. 2006). In this approach, it is supposed that VR serves as a facilitative tool, increasing individuals' ability to enter a hypnotic state. Nevertheless, current evidence does not support a clear superiority of VRH over standard VR or hypnosis to reduce pain. Rousseaux et al. (2022), in a study conducted in a cardiac surgery ICU setting, found that VRH was not significantly more effective than VR or hypnosis alone in reducing postoperative pain. Despite its promise, research directly comparing VR, hypnosis and VRH remains limited (Rousseaux et al. 2022; Wiechman et al. 2022). Rigorous studies incorporating both subjective (pain ratings) and objective (physiological measures) outcomes are necessary to clarify the comparative efficacy of these approaches and to further elucidate their underlying psycho‐physiological mechanisms (Rousseaux et al. 2020, 2023; Louras et al. 2024).

## 2 Methods

### 2.1 Objectives

Hypnosis and VR present valuable tools in pain management. However, understanding the potential synergistic effects of combining these techniques and their impact on pain outcomes requires further exploration through robust psycho‐physiological empirical research. This experimental study aimed to compare the effects of hypnosis, VR and VRH on the perception of pain intensity and unpleasantness and on the NFR.

### 2.2 Participants

A convenience sample of healthy participants was recruited through posters at the *Centre de recherche de l'Institut universitaire de gériatrie de Montréal* (CRIUGM) and via social media. To be eligible, volunteers had to (1) be healthy adults (> 18 years old). Exclusion criteria were as follows: (2) chronic pain not interfering with acute experimental pain, (3) diabetes (hyperglycemia can influence NFR), (4) neurological or psychiatric disorder, (5) psychotropic or analgesic medication and (6) claustrophobia or water‐related phobia (see VR below). Participants completed a preliminary questionnaire to ensure their eligibility.

### 2.3 Ethics

The participants gave their written consent and were compensated at a rate of 15 $CAD per hour for their time, which amounts to approximately 45 $CAD for their total participation. Ethical approval for this study was provided by the Comité d'évaluation scientifique de l'Université de Montréal (CÉS) (number: CER VN 21–22‐27) on October 20th 2021.

### 2.4 Procedure

There are a total of two sessions: (1) calibration and (2) intervention session involving all participants across all conditions. Before their first visit, participants had to complete consent and demographic data. During the first session, individual pain threshold was assessed. Participants were also invited to test the VR equipment to ensure that they did not experience nausea or dizziness. Virtual reality hypnosis was provided using a head‐mounted display. This device is composed of a smartphone plugged into a VR headset, an audio headset and integrated software (Medical device Class IA), forming the Oncomfort Sedakit X2 (HypnoVR SAS, Strasbourg, France, https://hypnovr.io/fr).

**FIGURE 1:**
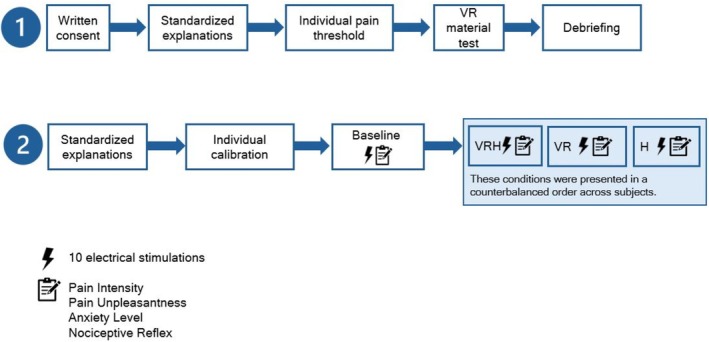
Procedure of the study.

The second visit began with a calibration to validate the individual pain threshold. Participants were then administered the baseline control condition followed by the three experimental conditions in a randomised order: hypnosis (H), virtual reality (VR), virtual reality hypnosis (VRH). To prevent a carry‐over effect affecting baseline measures, the first condition was always the baseline control, where participants received the stimuli without any intervention. Then the three experimental conditions were presented in a counterbalanced order between participants, to avoid a potential order effect. For each condition, participants received 10 painful electrical stimuli and were then asked to rate the intensity and unpleasantness of the pain. There was a washout of 15 min without any stimulation after each condition (Figure 1).

### 2.5 Conditions

**FIGURE 2:**
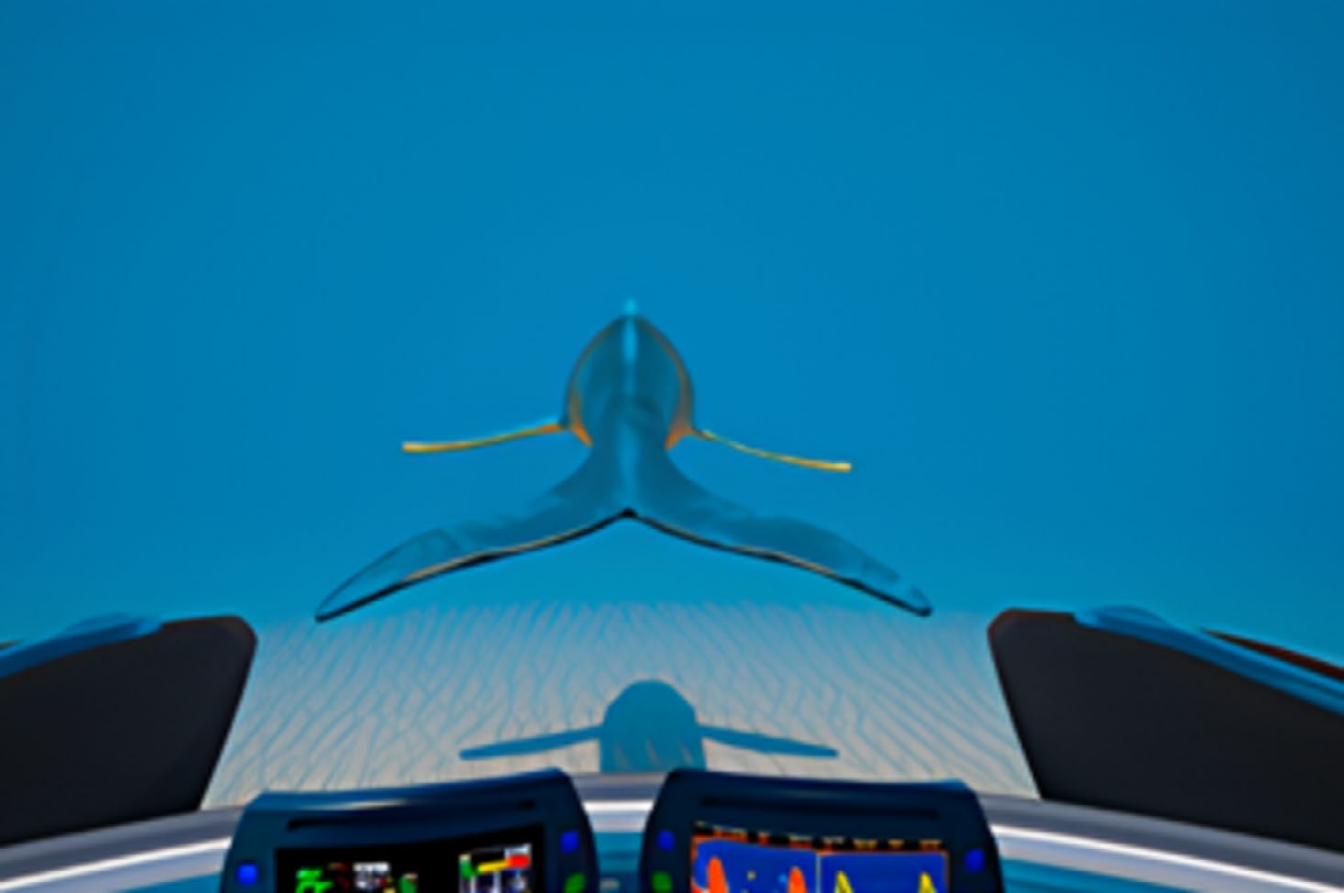
Example of a scene that participants viewed during the Aqua VRH experiment.

Conditions of the intervention session:

- *Baseline*: condition without any intervention.
- *Virtual reality (VR)*: viewing a relaxing underwater virtual environment without any audio.
- *Recorded hypnosis (H)*: listening to an audio recording of a hypnotic induction (~5 min) followed by guided relaxation and comfort suggestions (~10 min), without suggestions related to pain. Subjects were asked to keep their eyes closed during this condition. The hypnotic induction was based on progressive relaxation and the intervention included standard hypnotic phases (induction, deepening, suggestion and return) based on Aqua hypnotic scenario.
- *Virtual reality hypnosis (VRH)*: viewing the same relaxing underwater environment as the VR condition, while listening to the same recorded hypnosis session as the H condition. The Aqua VRH is a 16‐min session of an immersive virtual experience that combines a virtual 3D video with music, sounds and/or voice recording based on a hypnotic scenario recorded in French by a trained hypnotherapist. Visual experience is carefully synchronised with the hypnotic script and allows participants to induce and maintain a relaxed state with a disconnection from their external surroundings. The Aqua VRH session follows the standard phases of hypnosis. During the induction phase (3.52 min), subjects are over the surface of the sea. They are invited to focus their attention on their breath, and to induce progressive relaxation in their body. The guidance phase (1.24 min) brings the subjects slowly under the water. During the deepening phase (10.44 min), subjects follow a soothing underwater experience with specific suggestions regarding their comfort and relaxation. The re‐altering phase (1 min) brings the subjects progressively back into reality, concomitant with return to normal body sensations (Figure 2).

### 2.6 Assessments

#### 2.6.1 Acute Experimental Pain

The skin behind the left malleolus was cleansed using alcohol swabs, body hair was removed using a single‐use razor and skin was prepared with an abrasive cream to reduce impedance below 10 kohm. Reusable surface electrodes (EL258RT: 1 cm^2^, 2 cm inter‐electrode distance; BIOPAC Systems Inc., USA) were applied on the retromalleolar path of the left sural nerve with conductive gel. Painful transcutaneous electrical stimulation was delivered using a Digitimer DS7A constant current stimulator controlled by a BIOPAC MP150 system. The electrical stimuli consisted of a 20‐ms train of 5 × 1‐ms pulses at 200 Hz delivered with an inter‐train interval of at least 6 s.

#### 2.6.2 Pain Intensity and Pain Unpleasantness

Participants reported their pain unpleasantness and intensity ratings on a numerical rating scale (NRS) ranging from 0 to 100 (0 = no pain and 100 = most intense/unpleasant pain imaginable).

#### 2.6.3 Individual Pain Threshold

The threshold determination procedure involved the staircase method (Willer and Bathien 1977), where stimulation began at an imperceptible level and was gradually increased by 1 mA until the participant could detect a sensation (detection threshold). The stimulus intensity was then further increased until the participant felt pain (pain threshold), and then until an intensity was reached that elicited a NFR response. Subjective pain ratings were collected during the study to ensure that the stimuli were painful but always tolerable and acceptable in the context of a pain study.

The intensity of the stimuli was adjusted individually at 120%–135% of the participant's NFR reflex threshold, following methods previously used (Rhudy and France 2011). This intensity was determined to ensure that a reliable NFR reflex response was evoked, characterised by a clear response recorded in the EMG signal of the biceps femoris, and that the stimuli produced a mild to moderate pain level, allowing for the detection of any changes in pain perception. To maintain consistency, stimuli were then administered at that individually adjusted level for the experiment.

These measures were acquired with electrodes connected to the BIOPAC MP150 system.

#### 2.6.4 Nociceptive Flexion Reflex (NFR)

The flexion reflex was measured in response to each electrical stimulus via EMG, using a pair of EL508 surface electrodes placed on the shaved and exfoliated skin over the distal part of the short head of the biceps femoris. A ground electrode was also placed on the left tibial tuberosity. The EMG signal was full‐wave rectified and integrated (10 ms) and the resulting signal was used to quantify NFR amplitude to each shock by extracting the integral value between 90 and 150 ms after the stimulus onset (Willer and Bathien 1977; Sandrini et al. 2005; Willer and Albe‐Fessard 1983). To normalise the data across individuals, NFR amplitude values were transformed into T‐scores. This standardisation was performed by calculating the mean and standard deviation of each participant's raw NFR amplitudes and converting individual trial values using the formula: *T* = [(*X*—μ)/*σ*] × 10 + 50, where *X* is the raw score, μ is the participant's mean and *σ* is the participant's standard deviation. This transformation allows for meaningful comparison of reflex magnitudes both within and across subjects (Arsenault et al. 2013; Marouf et al. 2015).

### 2.7 Statistical Analysis

#### 2.7.1 Sample Size Estimation

The proposed sample size is based on previous studies. Within‐subject changes in acute pain responses (ratings) after hypnotic interventions have been reported to be in the moderate to large range, detectable with sample sizes as small as 16 participants (Thompson et al. 2019).

#### 2.7.2 Conditions Comparison

To investigate the comparative benefits of VRH over standalone VR and hypnosis on nociceptive processing and pain perception, we performed pairwise comparisons across four conditions: baseline, VR, H and VRH. We assessed differences between these conditions using paired samples t‐tests and quantified the evidence for our contrasts with Bayes Factors (BFs), computed using various widths of Cauchy priors via JASP software (JASP, n.d.).

Using *Bayes Factors (BFs)* to quantify the strength of evidence for the effects of hypnosis, VR and VRH on pain and nociceptive reflexes, while avoiding the limitations of frequentist tests and binary interpretation of *p* values. Additionally, by varying the Cauchy prior width, the robustness of the results is tested, ensuring that the conclusions are not overly dependent on subjective or restrictive prior assumptions.

Our analysis began by contrasting hypnosis, VR and VRH against baseline. We posited unidirectional hypotheses compared to baseline, anticipating that hypnosis, VR and VRH would elicit a reduction in the amplitude of the NFR and lower self‐reported pain intensity, and unpleasantness compared to baseline: H_0_: *δ* = 0 and H+: *δ* > 0, where *δ* is the standardised effect size, H_0_ represents the null hypothesis and H+ represents the alternative hypothesis. Moreover, we adopted bidirectional hypotheses while comparing hypnosis, VR and VRH. Based on Bayes Factor (BF) values, the strength of evidence in favour of our hypotheses was categorised following the guidelines proposed by Lee and Wagenmakers (2014): BF = 1–3: anecdotal evidence; BF = 3–10: moderate evidence and BF > 10: strong evidence.

The Bayesian approach was preferred over the frequentist approach (i.e., significance testing using *p* values) because it provides a continuous measure of evidence and allows quantifying support for both the null (H0) and the alternative hypothesis (H1), thereby overcoming a key limitation of *p* values. Compared to significance testing with *p* values, the Bayesian framework provides the following advantages: it yields a continuous and interpretable measure of evidence rather than relying on an arbitrary threshold; it allows quantifying support not only for the alternative hypothesis (H1) but also for the null hypothesis (H0); it is less sensitive to sample size inflation that can render trivial effects ‘significant’; and it enables the integration of prior theoretical or empirical knowledge into the analysis (Dienes and Mclatchie 2018; De Pascalis et al. 2008).

## 3 Results

### 3.1 Participants

**FIGURE 3:**
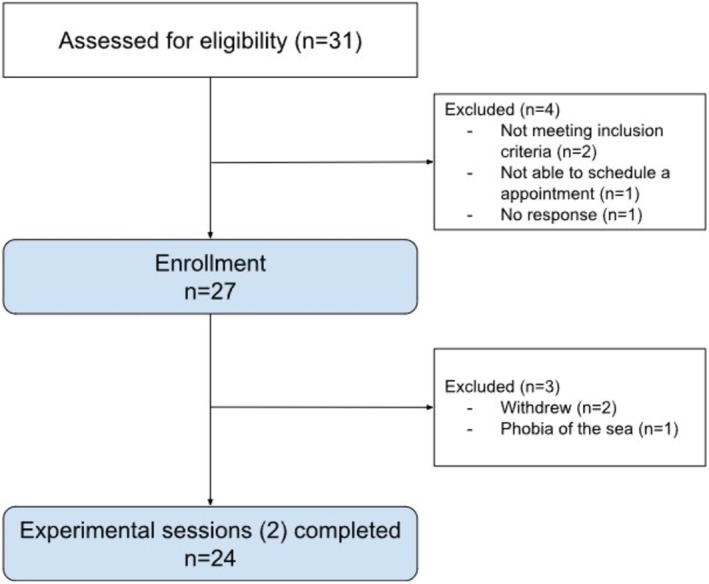
Flowchart of the participants.

Out of 31 participants screened, 24 healthy adults were included: 13 women (54.2%) and 11 men (45.8%). The mean age of the participants was about 30.8 years (±6). The majority had completed a university degree (*n* = 21), and a smaller proportion had completed a college (*n* = 1) or professional degree (*n* = 2) (Figure 3).

### 3.2 Subjective Report of Pain Intensity

**FIGURE 4:**
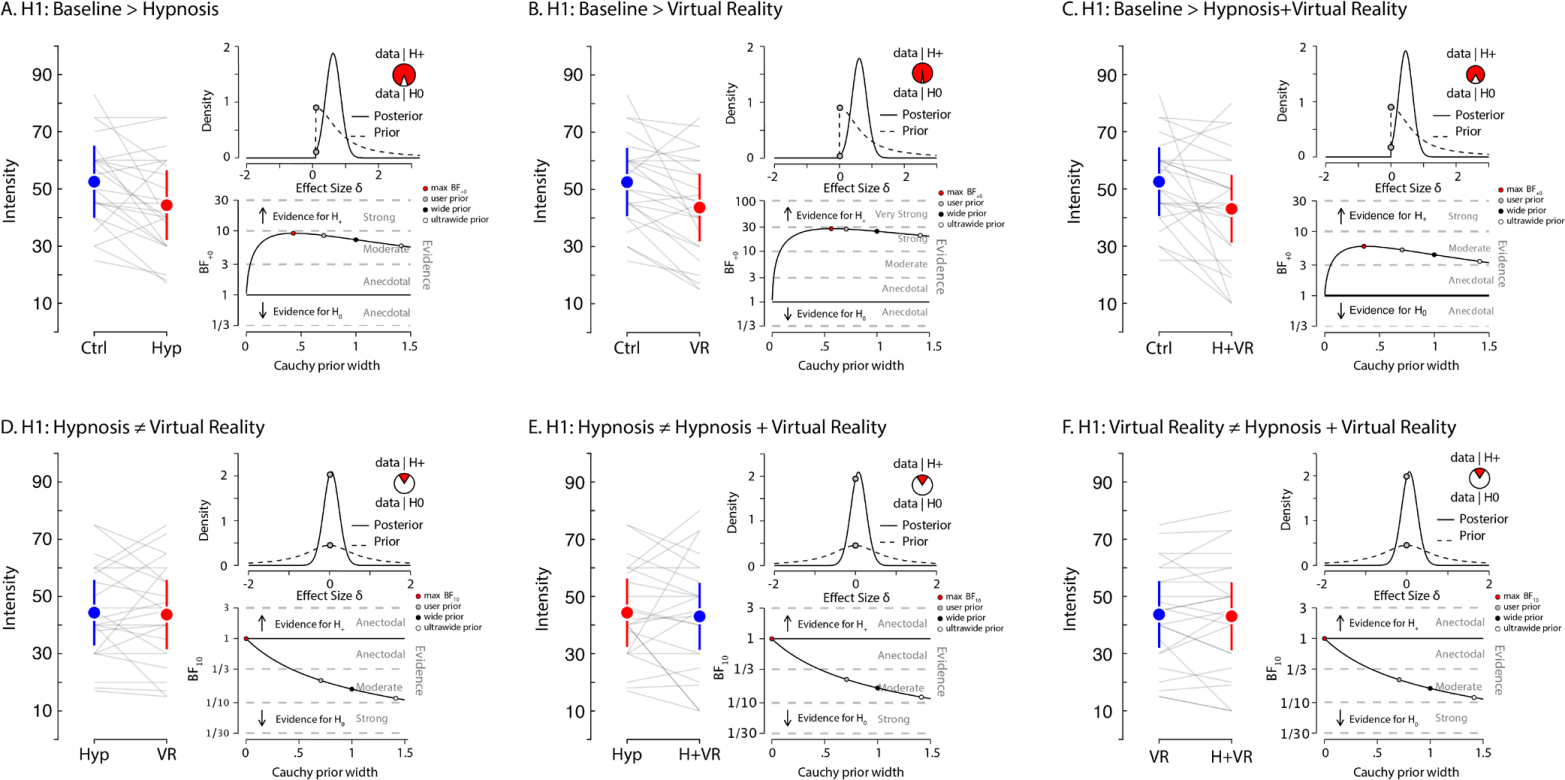
Pain intensity. Each panel presents the comparison between two conditions for the self‐reported measure of intensity of pain. For each panel, the left graph displays the average scores for both conditions, with error bars representing 95% C.I. Grey lines indicate individual participant values between conditions. In the upper‐right of each panel, the density plot illustrates the prior and posterior distributions of the estimated effect size (*δ*) for hypothesis H₁. This includes the likelihood of H₁ (the alternative hypothesis) compared to H₀ (the null hypothesis) based on the data. The lower‐right plot provides a robustness check of the Bayes factor (BF) analysis, depicting BF values as a function of different Cauchy prior widths. It highlights the maximum BF value, as well as the BF values for the user‐defined Cauchy prior (0.707), wide prior and ultra‐wide prior. Panel A presents the contrast where we hypothesized that the baseline control condition would exhibit greater feelings of pain intensity compared to the hypnosis condition. Panel B presents the contrast for our hypothesis that the baseline control condition would result in greater feelings of pain intensity than the VR condition. Panel C shows the hypothesized contrast that the baseline control condition would produce greater feelings of pain intensity compared to the combined hypnosis and VR (VRH) condition. For panel D, we hypothesized a difference in feelings of pain intensity values between the hypnosis and VR conditions. Panel E examines our hypothesis that there would be a difference in feelings of pain intensity values between hypnosis and the VRH condition. Panel F shows the evaluation for the hypothesis that there is a difference in feelings of pain intensity values between the VR and VRH conditions.

In the same vein, we hypothesized (H+) that self‐reported measures of pain intensity values would be higher during the baseline control condition compared to hypnosis, VR and VRH (Figure 4). A Bayesian paired‐sample T test provided moderate evidence supporting this hypothesis when comparing baseline with hypnosis (max BF_+0_ = 9.18 for a Cauchy prior width of 0.43; median *δ* = 0.51, 95% CI [0.12, 0.93]), indicating that hypnosis reduced pain intensity. In turn, the evidence for H+ was strong when comparing baseline control to VR (max BF_+0_ = 27.99 for a Cauchy prior width of 0.57; median *δ* = 0.62, 95% CI [0.2, 1.06]). The contrast between baseline and VRH also yielded moderate evidence in support of our hypothesis (max BF_+0_ = 5.88 for a Cauchy prior width of 0.37; median *δ* = 0.46, 95% CI [0.09, 0.88]). Again, when we investigated whether hypnosis, VR and VRH differentially impacted subjective reports of pain intensity, the evidence moderately favoured the null hypothesis across all comparisons: hypnosis versus VR (BF_10_ = 0.22 for a Cauchy prior width of 0.707; median *δ* = 0.05, 95% CI [−0.33, 0.43]); hypnosis versus VRH (BF_10_ = 0.23 for a Cauchy prior width of 0.707; median *δ* = 0.07, 95% CI [−0.3, 0.45]) and VR versus VRH (BF_10_ = 0.23 for a Cauchy prior width of 0.707; median *δ* = 0.06, 95% CI [−0.31, 0.44]). Consistent with our hypothesis, self‐reported pain intensity tended to be lower in experimental conditions (hypnosis, VR and VRH) compared to the baseline control, with moderate to strong Bayesian evidence supporting this effect, particularly for VR and hypnosis. However, contrasts between the experimental conditions suggest comparable effects of the three interventions on perceived pain intensity (Figure 4).

### 3.3 Subjective Report of Pain Unpleasantness

**FIGURE 5:**
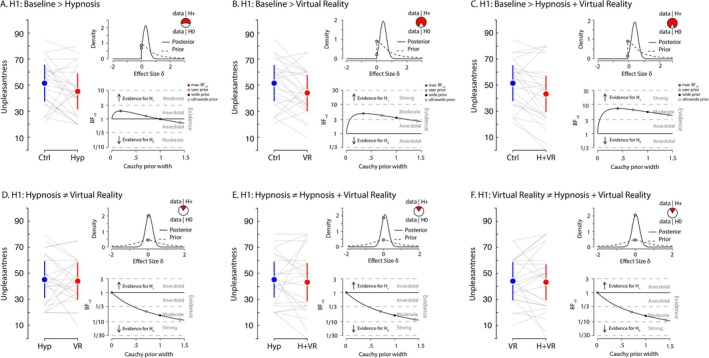
Pain unpleasantness. Each panel presents the comparison between two conditions for the self‐reported measure of unpleasantness of pain. For each panel, the left graph displays the average scores for both conditions, with error bars representing 95% C.I. Grey lines indicate individual participant values between conditions. In the upper‐right of each panel, the density plot illustrates the prior and posterior distributions of the estimated effect size (*δ*) for hypothesis H₁. This includes the likelihood of H₁ (the alternative hypothesis) compared to H₀ (the null hypothesis) based on the data. The lower‐right plot provides a robustness check of the Bayes factor (BF) analysis, depicting BF values as a function of different Cauchy prior widths. It highlights the maximum BF value, as well as the BF values for the user‐defined Cauchy prior (0.707), wide prior and ultra‐wide prior. Panel A presents the contrast where we hypothesized that the baseline control condition would exhibit greater feelings of pain unpleasantness compared to the hypnosis condition. Panel B presents the contrast for our hypothesis that the baseline control condition would result in greater feelings of pain unpleasantness than the VR condition. Panel C shows the hypothesized contrast that the baseline control condition would produce greater feelings of pain unpleasantness compared to the combined hypnosis and VR (VRH) condition. For panel D, we hypothesized a difference in feelings of pain unpleasantness values between the hypnosis and VR conditions. Panel E examines our hypothesis that there would be a difference in feelings of pain unpleasantness values between hypnosis and the VRH condition. Panel F shows the evaluation for the hypothesis that there is a difference in feelings of pain intensity values between the VR and VRH conditions.

We also evaluated these hypotheses for the self‐reported measure of unpleasantness (Figure 5). We hypothesized (H+) that unpleasantness would be greater during the baseline control condition compared to hypnosis, VR and VRH. The analysis provided anecdotal support for this hypothesis when comparing baseline with hypnosis (max BF + 0 = 1.9 for a Cauchy prior width of 0.18; median *δ* = 0.31, 95% CI [0.03, 0.7]), indicating that the effect of hypnosis on reducing unpleasantness was relatively limited. In contrast, the evidence for H+ was moderate when comparing baseline control to VR (max BF + 0 = 4.86 for a Cauchy prior width of 0.35; median *δ* = 0.43, 95% CI [0.08, 0.85]) and to VRH (max BF + 0 = 7.18 for a Cauchy prior width of 0.41; median *δ* = 0.48, 95% CI [0.1, 0.9]). Furthermore, when examining whether hypnosis, VR and VRH differentially affected the subjective reports of pain unpleasantness, the evidence moderately favoured the null hypothesis across all comparisons: hypnosis versus VR (BF10 = 0.24 for a Cauchy prior width of 0.707; median *δ* = 0.06, 95% CI [−0.32, 0.44]); hypnosis versus VRH (BF10 = 0.24 for a Cauchy prior width of 0.707; median *δ* = 0.09, 95% CI [−0.29, 0.47]) and VR versus VRH (BF10 = 0.22 for a Cauchy prior width of 0.707; median *δ* = 0.04, 95% CI [−0.33, 0.42]). In line with our hypothesis, subjective unpleasantness ratings tended to be lower in the experimental conditions (hypnosis, VR and VRH) compared to the baseline control. Bayesian analyses provided anecdotal evidence for a reduction with hypnosis, and moderate evidence for reductions with VR and VRH. However, contrasts among the experimental conditions yielded moderate evidence suggesting comparable effects on unpleasantness (Figure 5).

### 3.4 Nociceptive Flexion Reflex

**FIGURE 6:**
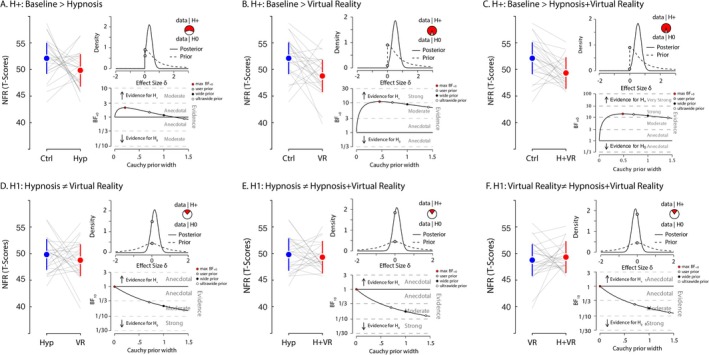
Nociceptive flexion reflex. Each panel presents the comparison between two conditions for the NFR measure. For each panel, the left graph displays the average scores for both conditions, with error bars representing the 95% C.I. Grey lines indicate individual participant values between conditions. In the upper‐right of each panel, the density plot illustrates the prior and posterior distributions of the estimated effect size (*δ*) for hypothesis H₁. This includes the likelihood of H₁ (the alternative hypothesis; in red on the graph) compared to H₀ (the null hypothesis, in white on the graph) based on the data. The lower‐right plot provides a robustness check of the Bayes factor (BF) analysis, depicting BF values as a function of different Cauchy prior widths. It highlights the maximum BF value, as well as the BF values for the user‐defined Cauchy prior (0.707), wide prior and ultra‐wide prior. Panel A presents the contrast where we hypothesized that the baseline control condition would exhibit greater nociceptive flexion reflex (NFR) compared to the hypnosis condition. Panel B presents the contrast for our hypothesis that the baseline control condition would result in greater NFR than the VR condition. Panel C shows the hypothesized contrast that the baseline control condition would produce greater NFR compared to the combined hypnosis and VR (VRH) condition. For panel D, we hypothesized a difference in NFR values between the hypnosis and VR conditions. Panel E examines our hypothesis that there would be a difference in NFR values between hypnosis and the VRH condition. Panel F shows the evaluation for the hypothesis that there is a difference in NFR values between the VR and VRH conditions.

We hypothesized (H^+^) that NFR values would be lower during the experimental conditions hypnosis, virtual reality (VR) and (VRH) compared to the baseline control condition. A Bayesian paired‐sample T test provided anecdotal evidence supporting this hypothesis when comparing baseline with hypnosis (max BF_+0_ = 2.11 for a Cauchy prior width of 0.19; median *δ* = 0.33, 95% CI [0.04, 0.72]), suggesting that the benefit of hypnosis in reducing NFR was relatively modest. In contrast, the evidence for H+ was stronger when comparing baseline control to virtual reality (max BF_+0_ = 10.94 for a Cauchy prior width of 0.46; median *δ* = 0.53, 95% CI [0.13, 0.96]). Similarly, the contrast between baseline and VRH also yielded strong evidence in support of our hypothesis (max BF_+0_ = 14 for a Cauchy prior width of 0.49; median *δ* = 0.55, 95% CI [0.15, 0.98]). On the other hand, when we investigated whether hypnosis, VR and VRH differentially affected the NFR, the evidence moderately favoured the null hypothesis across all comparisons: hypnosis versus VR (BF_10_ = 0.3 for a Cauchy prior width of 0.707; median *δ* = 0.16, 95% CI [−0.22, 0.54]); hypnosis versus VRH (BF_10_ = 0.25 for a Cauchy prior width of 0.707; median *δ* = 0.1, 95% CI [−0.28, 0.48]); and VR versus VRH (BF_10_ = 0.25 for a Cauchy prior width of 0.707; median *δ* = −0.1, 95% CI [−0.48, 0.28]). The results suggest that all three experimental conditions—hypnosis, VR and VRH—were associated with reductions in NFR amplitude relative to the baseline control condition, with stronger evidence observed for VR and VRH compared to hypnosis alone; however, there was only modest or anecdotal support for differences among the experimental conditions themselves (Figure 6).

## 4 Discussion

The aim of this study is to report on which technique (hypnosis, VR, VRH) is best able to reduce the experimental pain response assessed using self‐reports of pain intensity and unpleasantness and physiological mechanisms underlying each of these complementary approaches.

Regarding subjective measures, the study demonstrated reductions in both pain intensity and unpleasantness across all experimental conditions (hypnosis, VR and VRH) as compared to the baseline. More specifically, VR and VRH showed stronger evidence of reducing pain intensity compared to hypnosis alone, with moderate to strong Bayesian support for these effects, particularly for VR. Moreover, the comparison between VR and hypnosis showed moderate evidence for equivalence in their effects on pain intensity and unpleasantness, although these results were influenced by substantial individual variability. This apparent equivalence may also be partially explained by potential task‐related attentional effects, which may have underestimated the impact of hypnosis. Taken together, these findings support the notion that immersive VR can be particularly effective in reducing pain perception, as indicated in previous research (Mallari et al. 2019), though further investigation is needed to fully understand the interplay of these experimental conditions on both subjective pain experiences and physiological measures. Potential mediators of treatment effects may include attentional focus, emotional regulation, immersion levels and expectations regarding the intervention. These mechanisms may differentially explain how VR, hypnosis and VRH act on the sensory and affective dimensions of pain.

Turning to physiological measures, the VR and VRH conditions show large evidence for reduction in reflex amplitude compared to baseline. In contrast, when comparing the hypnosis condition to either VR or VRH, no evidence of differential effects on reflex amplitude is observed. Some participants exhibited increased reflex amplitudes during hypnosis, suggesting that the focused attention required for hypnosis might paradoxically heighten reflex responses in certain individuals, a finding supported by a study on the variability of hypnotic effects (Thompson et al. 2019; Danziger et al. 1998). These findings are supported by Terzulli et al. (2022, 2023) who demonstrated that VRH increases thermal pain thresholds, reduces cold pain perception and modulates both cortical and autonomic responses, confirming its effects on subjective pain and physiological markers. This study adds further evidence that VRH can modulate both the sensory and autonomic components of pain, highlighting its potential as a multifaceted tool for pain management.

Furthermore, our study highlighted that VR alone had an effect on reducing both pain intensity and unpleasantness, with little to no additional benefit when hypnosis was added to the VR condition. Kenney and Milling (2016) established that distractive virtual reality reduces experimental pain more effectively than clinical pain, suggesting that the effectiveness of VR in pain management may vary depending on the pain context (Kenney and Milling 2016). Montgomery et al. (2000) found that hypnosis is similarly effective in reducing both experimental and clinical pain, implying that the mechanisms involved in pain management with either technique differ. More recently, Cardinal et al. (2024) found that VRH may help reduce experimental pain but noted potential interference between VR and hypnosis when cognitive load is high (Cardinal et al. 2024). In this study, the relaxing nature of the VR content may have reduced contrasts between the active interventions. This limitation can be noted as a potential confounder. Some people have reported a feeling of sensory saturation, a discomfort felt in the attempt to engage both in the visual environment and with the suggestions of the audio script. In such cases, the two procedures may, instead of enhancing one another, interfere with each other (Enea et al. 2014). This idea aligns with prior findings by Patterson et al. (2006), who observed that combining hypnosis with virtual reality for pain management did not outperform either technique used separately. In fact, their results raised the possibility that using both simultaneously might dilute the unique attentional mechanisms each method relies on—hypnosis demanding inward focus, and VR encouraging external visual engagement. All three interventions shared relaxation components by design, which allowed us to compare their foundational mechanisms under controlled conditions. This should not be viewed as a limitation but rather as a methodological strength, since it reduced potential confounds associated with complex VR tasks or additional analgesic suggestions. In a subsequent study (Patterson et al. 2006), they found that a sequential design, where hypnosis was used to enhance VR distraction (rather than applied simultaneously), produced superior analgesic effects. This finding suggests that using hypnosis to prime attentional and imaginative capacities before a VR session could represent a more effective integrative approach. An additional possibility is that VRH could represent a hybrid experience, combining immersive visual stimuli that capture attention with imaginative suggestion‐based processes more traditionally associated with eyes‐closed hypnosis. More broadly, the inconsistent outcomes in VRH research highlight unresolved questions regarding its optimal design—whether it should primarily be relaxing, distractive, or a hybrid experience. Furthermore, hypnotizability appears to be a key moderator.

In a clinical context, a prospective randomised trial by Rousseaux et al. (2022) evaluated the effectiveness of combining VR and hypnosis for managing anxiety and pain in cardiac surgery patients in intensive care units. The results showed that in this context, none of the techniques demonstrated a significant difference compared to the control group. Additionally, Carella et al. (2025) explored the impact of VRH during knee arthroplasty under spinal anaesthesia. Their findings demonstrated that patients in the VRH group required significantly less intraoperative sedation and experienced improved postoperative functional recovery compared to the control group. This suggests that VRH may serve as a valuable adjunct for perioperative pain and anxiety management in surgical settings, offering both clinical and functional benefits. In summary, while VRH demonstrates potential in reducing experimental and clinical pain, its effectiveness appears to depend heavily on the context of use and individual characteristics. VR alone shows strong and consistent effects on pain intensity and unpleasantness, whereas the added benefit of combining it with hypnosis remains uncertain. Therefore, although VRH may offer promising benefits in certain settings—especially perioperative contexts—more targeted research is needed to clarify its mechanisms, define optimal usage conditions and develop practical guidelines for clinical implementation.

In this study, the hypnotic script used in both the H and VRH conditions was identical and focused on relaxation and breathing. The same audio recording was used in both interventions to ensure consistency across conditions. Nevertheless, several limitations of this study must be considered. First, the hypnotic script focused on breathing and relaxation rather than pain reduction, potentially reducing its analgesic efficacy. This may account for its weak to moderate effects, as previous research has indicated that adding direct or indirect hypoalgesic suggestions to hypnotic induction is important to achieve moderate to large pain relief (Thompson et al. 2019; De Pascalis et al. 2008). Furthermore, the use of standardised VR equipment posed another limitation. Technical issues, including low image resolution, may have diminished the effectiveness of the VRH protocol; thus, higher‐quality VR content, such as more immersive landscapes, could potentially enhance outcomes (Garcia‐Palacios et al. 2001). The experimental design also exhibited constraints. Baseline measurements were consistently taken at the start of sessions, potentially masking certain effects. As our sample size estimation focused primarily on detecting within‐subject differences between baseline and each intervention, we may not have sufficient statistical power to detect potentially smaller differences between the treatments (VR vs. VRH). Additionally, the sample consisted of relatively young and healthy individuals, which limits the generalizability of our findings to broader, more diverse or clinical populations. It also primarily comprised psychology students, which introduces a selection bias; these individuals are typically familiar with the studied psychological concepts and possess a high educational level. This population does not accurately represent a typical hospital patient demographic, which includes various pain types, serious pathologies, anxiety and fatigue. It is important to note that the acute pain in this study was induced experimentally via electric shocks, which do not reflect pain related to pathology.

For future perspectives, we suggest that virtual reality hypnosis techniques should be further developed, whereby the virtual environment and hypnotic suggestions targeting pain are integrated rather than simply superimposed. Furthermore, improvements to the VR and VRH programs regarding image quality are warranted, as such factors have been shown to influence analgesic effectiveness (Hoffman et al. 2006). Testing different types of programs in subsequent studies could yield valuable insights. The mechanisms and effectiveness of VRH should be evaluated on a case‐by‐case basis, considering the individual needs and characteristics of patients, as well as the specific clinical contexts and purposes (Rousseaux et al. 2020). For example, VRH may be particularly beneficial for individuals who are new to hypnosis, those who have difficulties with visualisation and those looking to expedite their learning process (Schnur et al. 2008). Further research is needed to understand whether participants can enhance their hypnosis skills or susceptibility through VRH. Future studies should explore this effect more comprehensively, using larger sample sizes and more precise measures. Another important limitation is that we did not correlate our results with participants' hypnotizability. This variable is known to strongly influence responsiveness to hypnotic suggestions, particularly in the context of pain modulation (Jensen et al. 2015). Without measuring hypnotic susceptibility, it is difficult to determine whether the limited efficacy of the hypnosis or VRH conditions was due to the intervention itself or to individual differences in suggestibility. Including a standardised measure of hypnotizability in future studies would allow for more precise interpretation of the results and may help identify which subgroups are most likely to benefit from hypnosis‐based interventions. More broadly, the unresolved questions in VRH research suggest the need to determine whether its optimal design should emphasise relaxation, distraction or hybrid approaches. In addition, sequential protocols in which hypnosis primes VR distraction may represent a particularly promising direction. Furthermore, integrating biofeedback or neurofeedback into VRH environments could dynamically adapt the experience to the user's physiological state, potentially enhancing both immersion and analgesic effectiveness.

Ultimately, understanding whether VRH can be optimised for pain management or if the combination of these techniques inhibits their efficacy remains a key goal for both research and clinical practice (Louras et al. 2024). It is important to clarify whether combining hypnosis and VR provides additive or synergistic effects. Looking to the future, there is a clear priority to develop more personalised pain management approaches that consider individual differences. Besides, we need more studies that contrast a verbal hypnotic induction, with one that involves the visual stimulation inherent in VRH. Moreover, one intriguing but underexplored possibility is the integration of physiological feedback—such as biofeedback or neurofeedback—into VRH systems. Real‐time adjustments to virtual stimuli based on the user's physiological state (e.g., heart rate, EEG patterns) could help optimise the hypnotic or analgesic effect dynamically. Future studies could explore the potential of such feedback‐driven environments to deepen engagement and personalise treatment.

Future research should investigate how the ability to dissociate or immerse oneself in VRH influences responses to these techniques (Mallari et al. 2019). Comparing the mediation models of VR and hypnosis may provide insights into how these techniques differentially influence the sensory and emotional components of pain, potentially enhancing pain management strategies (Enea et al. 2014; Melzack and Wall 1965), such as attentional focus, emotional regulation, immersion levels or expectations about the intervention. These variables could be assessed using psychometric tools or physiological markers. Clinical studies in specialised contexts, such as intensive care, burn pain and oncology, where VRH could be used for distraction during painful invasive procedures and hypnosis could assist in self‐management of pain, present promising future applications (Safy et al. 2024). Long‐term studies with larger sample sizes will be essential to confirm the current findings and explore how these techniques can be effectively employed in managing acute and chronic pain in clinical situations (Wiederhold et al. 2014).

In conclusion, this study suggests that VR is a promising approach for pain management, and the addition of hypnosis does not consistently enhance the effects, and in some cases, may introduce complexity that requires further investigation. While these techniques may operate through partly different mechanisms, they might also interfere with one another if not adequately integrated. Further research on psychological mechanisms is necessary to better understand the interplay between VR and hypnosis and to optimise their use in personalised pain management strategies.

## Author Contributions

A.V. and P.R. are guarantors. Design and conception of the study: F.R., A.V., P.R. Project supervisors: A.V. and P.R. Acquisition of data: F.R., E.G., P.A. Material conception: M.‐E.F., D.J., HypnoVR Society. Statistical analysis: M.L., M.P., M.M. Interpretation of data: all authors. Writing up of the first draft: F.R., E.G. Revision of the draft: all authors. Revision of the final draft: all authors. Read and approved the final manuscript: all authors.

## Ethics Statement

Ethical approval for this study was provided by the Comité d'évaluation scientifique de l'Université de Montréal (CÉS) (number: CER VN 21–22‐27) on October 20th 2021. Written informed consents were obtained from all participants before inclusion. There is no plan to individually notify participants regarding the results of this study.

## Consent

All participants gave their signed consent to publication.

## Conflicts of Interest

The authors declare no conflicts of interest.

## Data Availability

Requests for access to data and statistical code should be addressed to FR or AV. FR, AV and PR guarantee that the manuscript is an honest, accurate and transparent account of the study being reported; that no important aspects of the study have been omitted; and that any discrepancies from the study as planned (and, if relevant, registered) have been explained.
